# GNAQ and BRAF mutations show differential activation of the mTOR pathway in human transformed cells

**DOI:** 10.7717/peerj.104

**Published:** 2013-07-23

**Authors:** Helena Pópulo, Sandra Tavares, Alexandra Faustino, Joana B. Nunes, José Manuel Lopes, Paula Soares

**Affiliations:** 1 Institute of Molecular Pathology and Immunology, University of Porto, Porto, Portugal; 2 Medical Faculty, University of Porto, Porto, Portugal; 3 Department of Pathology, Hospital São João, Porto, Portugal

**Keywords:** Melanoma, GNAQ, BRAF, mTOR, MAPK

## Abstract

Somatic mutations in *GNAQ* gene were described as being the main oncogenic activation in uveal melanomas, whereas mutations in *BRAF* gene have been described as a key genetic alteration that contributes to skin melanoma development. We have previously reported differential activation of the MAPK and AKT/mTOR signalling pathways in uveal and skin melanomas harbouring, respectively, GNAQ and BRAF mutations. The aim of this work was to compare the functional effect of GNAQ and BRAF mutations in mTOR and MAPK pathway activation, cell proliferation and apoptosis. In this work, we performed transient transfection of HEK293 cells with BRAF^WT^, BRAF^V 600E^, GNAQ^WT^, GNAQ^Q209P^ and GNAQ^Q209L^ vectors. We treated melanoma cell lines displaying different BRAF and GNAQ mutational status with the mTOR inhibitor RAD001 and with the MEK1/2 inhibitor U0126 and evaluated the effects in the growth of the cell lines and in mTOR and MAPK pathway effectors expression. At variance with the significant increase in the level of pmTOR Ser2448 and pS6 Ser235/236 proteins observed in cells transfected with BRAF vectors, no significant alteration in mTOR pathway effectors was observed in cells transfected with the three GNAQ expressing vectors. Also, GNAQ overexpression enhances Stat3 activation, which might mediate GNAQ oncogenic effects. None of the vectors led to significant differences in proliferation or apoptosis in the transfected cell lines. Cell lines harbouring a BRAF mutation were more sensitive to RAD001 treatment. U0126 leads to the reduction of MAPK and mTOR pathways activation in all cell lines tested. Our results indicate that GNAQ and BRAF activation drive distinct intracellular signalling pathways that may be useful for therapeutic decisions in human melanomas.

## Introduction

Melanoma arises from the malignant transformation of the melanocytes (review in Tolleson, 2005). The skin is the most common site for melanoma development, followed by the eye, although tumours arising on these two locations display different biological and clinical behaviours (Didolkar et al., 1980).

Ocular melanoma is the most common primary eye tumour in adults, and accounts for ∼5% of all melanomas. Most arise in the uvea with >90% occurring in the choroid and few arising in the ciliary body and iris, and <5% in the conjunctiva (Chang, Karnell & Menck, 1998). Skin melanoma represents <5% of all skin cancer but is responsible for the majority of skin cancer-related deaths (www.cancer.org). After metastasis, treatment options available for ocular and skin melanomas show limited efficacy (Damato, 2004; Gray-Schopfer, Wellbrock & Marais, 2007; Tarhini & Agarwala, 2006).

Melanomagenesis is thought to occur by the accumulation of several genetic and molecular alterations (Miller & Mihm, 2006; Wolchok & Saenger, 2007), some of which lead to the activation of the MAPK and AKT/mTOR signalling (Dahl & Guldberg, 2007).

Somatic mutations in BRAF gene have been described as key genetic alterations in skin melanoma development (Smalley & Herlyn, 2004), whereas GNAQ gene was described as an oncogene in uveal melanomas (Van Raamsdonk et al., 2009). Both alterations are likely to result in MAPK pathway activation.

In a previous work we reported BRAF mutations in 30% of skin melanomas and GNAQ gene mutations in 36% of uveal melanomas (Populo et al., 2011a; Populo et al., 2011b). No significant association was found between BRAF or GNAQ mutations and the expression of phosphorylated ERK1/2 in tumours, as previous reported by others for BRAF mutations (Houben et al., 2008). An association between BRAF mutation and elevated mTOR pathway activation was observed in skin melanomas, whereas in a series of uveal melanomas no association was found between mTOR pathway activation and GNAQ mutation (Populo et al., 2011a; Populo et al., 2011b). Our group also found in papillary thyroid carcinoma (that also presents frequent mutation in BRAF gene) an increased activation of mTOR pathway in BRAF mutated PTC, and *in vitro* transfection of BRAF^V 600E^ disclosed a positive association between BRAF (over)expression and mTOR pathway activation (Faustino et al., 2012). Inactivation of LKB1 by Ser428 phosphorylation might mediate the association between BRAF expression and mTOR pathway regulation (Faustino et al., 2012).

Our aim was to compare *in vitro* the effect of GNAQ and BRAF mutations in the activation of MAPK and mTOR pathways and in the sensitivity to the inhibition of those pathways.

## Material and Methods

### Cell lines and culture conditions

The BLM, G361 and Mewo skin melanoma cell lines were kindly provided by Dr. Marc Mareel, from the Department of Radiotherapy and Nuclear Medicine, Ghent University Hospital, Belgium. The A375 skin melanoma cell line was kindly provided by Dr. Madalena Pinto, from CEQUIMED, Faculty of Pharmacy, University of Porto, Portugal. 92.1 (De Waard-Siebinga et al., 1995), OMM1 (Luyten et al., 1996), OMM2.3 (Chen et al., 1997) and Mel285 (Ksander et al., 1991) uveal melanoma cell lines were kindly provided by Dr. Martine Jager, from the Laboratory of Ophthalmology, Leiden University, Netherlands. The HEK293 cell line was kindly provided by Dr. Bart Eggen, from the Department of Neuroscience, University of Groningen, Netherlands. HEK293 cells, derived from human embryonic kidney cells, were used as a model system to verify the effect of the overexpression/activation of BRAF and GNAQ genes in the expression of MAPK and mTOR pathways effectors. All the cell lines were tested for mycoplasma.

The BLM, Mewo and HEK293 cell lines were maintained in DMEM medium (Gibco/BRL – Invitrogen), the G361 cell line was maintained in McCoy’s medium (Gibco/BRL – Invitrogen), and the 92.1, OMM1, OMM2.3 and Mel285 cell lines were maintained in RPMI medium (Gibco/BRL – Invitrogen). All media were supplemented with 10% of fetal bovine serum, 100 U/mL Penicillin and 100 ug/mL Streptomycin. Cell lines were maintained in a humidified atmosphere (5% CO_2_) at 37°C.

### Expression vectors and cell transfection

The GNAQ^WT^ vector was purchased from UMR cDNA Resource Center. The mutant forms of GNAQ gene (GNAQ^Q209P^ and GNAQ^Q209L^) were generated by site-directed mutagenesis. All plasmids were re-sequenced to confirm that the desired mutations were introduced without changes to the vector backbone. The coding sequences of GNAQ^WT^, GNAQ^Q209P^ and GNAQ^Q209L^ were cloned into the expression plasmid pcDNA3.1.

Transient transfection of HEK293 cells was performed by the calcium phosphate co-precipitation method (Clark et al., 1995), 24 h after cells were seeded (1 × 10^5^/well) in 6-well plates. Cells were transfected with 5 ug of plasmid DNA, which included 500 ng of the expression plasmid (pcDNA3.1- GNAQ^WT^, pcDNA3.1- GNAQ^Q209P^ or pcDNA3.1- GNAQ^Q209L^) or pcDNA3.1- empty vector, 500 ng of pEGFP-C1 (Clontech, Mountain View, USA) to monitor transfection efficiency, and 4 ug of “carrier DNA”- pUC18. Confirmation of GNAQ increase expression as well as of pERK 1/2 (as a readout of GNAQ activity) was done by Western-blotting. BRAF wild-type and mutant vectors were obtained as described in Faustino et al., 2012.

### Treatment of melanoma cell lines with RAD001 and U0126

RAD001 (Everolimus, Novartis Pharma AG, Basel, Switzerland) was dissolved in DMSO and added to the culture medium. 20 nM and 50 nM of RAD001 were used for 24 and 48 h treatment. Melanoma cells incubated with culture medium supplemented with DMSO served as the control. U0126 (Sigma-Aldrich, St. Louis, MO, USA) was dissolved in DMSO and added to the culture medium. As recommended by the manufacturer, 20 µM of U0126 were used for 24 h of treatment.

### Western blot analysis and antibodies

Cells were lysed for 15 min at 4°C using RIPA buffer (1% NP-40 in 150 mM NaCl, 50 mM Tris (pH 7.5), 2 mM EDTA) containing phosphatase and protease inhibitors. Proteins were quantified using a modified Bradford assay (Biorad). Protein samples (50 µg) were separated in 6% or 12% SDS/PAGE gels, depending on the molecule to be analysed, and electroblotted to Hybond ECL membrane (Amersham Biosciences). We used the following primary antibodies: PTEN, phospho-mTOR Ser2448, phospho-AKT Ser473, phospho-S6 Ser235/236, phospho-4E-BP1 Thr37/46, phospho-ERK1/2 Thr202/Tyr204, raptor (all from Cell Signaling Technology), rictor (Abnova), and BRAF and GNAQ (Santa Cruz Biotechnology). Secondary antibodies were conjugated with peroxidase (Santa Cruz Biotechnology) and visualized by the ECL detection solution. Membranes were re-stained with a goat polyclonal anti-actin (Santa Cruz Biotechnology) antibody for loading protein control. All experiments and quantifications (using Bio-Rad Quantity One 1-D Analysis software (4.6.6 version)) were performed in triplicate.

### BrdU assay

Forty-eight hours after transfection, the cells seeded on coverslips were labelled by incubation in 10 µM of bromodeoxyuridine (BrdU) for 1 h and fixed with 4% paraformaldehyde. Nuclear incorporation was detected using an anti-BrdU antibody (Dako^®^). The proportion of positive nuclei (BrdU index) was determined counting at least 500 cells.

### TUNEL assay

Forty-eight hours after transfection, cytospin preparations of both floating and attached cells were collected. Cells were fixed with 4% paraformaldehyde at room temperature, washed in PBS and permeabilised with 0.1% Triton X-100 in 0.1% sodium citrate on ice. TUNEL analysis was performed using the “In situ cell death detection kit, fluorescein” from Roche^®^, following the manufacturer’s instructions. The proportion of TUNEL-positive nuclei was determined from counting at least 500 cells.

### Sulforhodamine B (SRB) assay

Cells were seeded as triplicates in 96-well plates at a density of 6 × 10^2^ for skin melanoma cell lines and 8 × 10^2^ for ocular melanoma cell lines in 200 µl medium. After 24 h, the medium was replaced by a medium containing 20 nM of RAD001. Cells were incubated for 24 and 48 h, fixed in 50 µl of cold 50% Trichloroacetic acid, washed with distilled water, and air dried. 150 µl of a SRB solution at 0.1% in 1% acetic acid was then added. The plates were incubated for 30 min at RT, washed with 1% acetic acid and air dried. Finally, 150 µl of 10 mM Tris-base was added, plates were shaken and measured at 560 nm, using a Synergy Mx microplate reader (BioTek Instruments, Inc., Winooski, VT). The intensity of absorbance indicates the number of viable cells in the wells (Skehan et al., 1990). The absorbance of the wells containing culture medium with DMSO and tumour cells was used as control, whereas culture medium with DMSO alone was used as blank calibration. Results were expressed as a percentage of the growth relative to the control. Each experimental condition was studied with triplicates and repeated in duplicate.

### DNA extraction and mutation analysis

DNA extraction was done using the Invisorb spin tissue mini kit (Invitek, Berlin). Fragments encompassing BRAF exon 15, NRAS exon 2 and GNAQ exon 5 were amplified by polymerase chain reaction (PCR). Genomic DNA (25–100 ng) was amplified by PCR using the following cycling conditions: 35 s at 94°C, 40 s at 58°C for BRAF and GNAQ and 57° for NRAS and 45 s at 72°C for 35 cycles. All PCR products were purified and directly sequenced on an ABI Prism 3130 *xl* Automatic sequencer (Perkin-Elmer, Foster City, CA) using the ABI Prism Dye Terminator Cycle sequencing kit (Perkin-Elmer).

### Statistical analysis

Statistical analysis was performed using STAT VIEW-J 5.0 (SAS Institute, Inc., Cary, NC). The data was analysed by the two-tailed unpaired Student’s *t*-test. A *p* value <0.05 was considered statistically significant.

## Results

### Expression of MAPK and mTOR pathways effectors in transfected cell lines with GNAQ vectors

**Figure 1:**
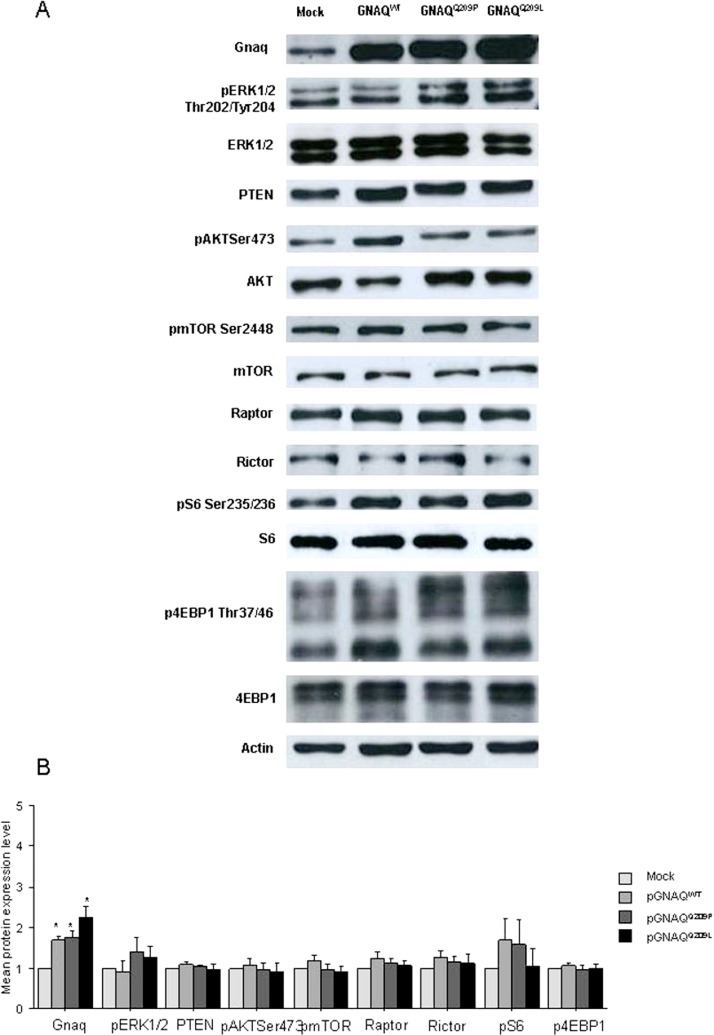
(A) Representative Western blot analysis of GNAQ, pERK1/2 and mTOR pathway effectors expression in HEK293 cells, transfected with GNAQ^WT^, GNAQ^Q209P^ and GNAQ^Q209L^ expressing vectors compared to cells transfected with an empty vector, in at least three sets of experiments; (B) graphic representation of the mean fold change of activated protein expression observed. Error bars are standard error. ∗ refers to a significant (*p* < 0.05) difference when comparing cells transfected with GNAQ^WT^, GNAQ^Q209P^ and GNAQ^Q209L^ with those with the empty vector.

The efficiency of transfection of HEK293 cells with GNAQ^wt^, GNAQ^Q209P^ and GNAQ^Q209L^ vectors was as high as 60% in all experiments, assessed by fluorescence microscope, and also observed by the levels of GNAQ expression and ERK1/2 activation, which was higher with the mutated vectors than with GNAQ^wt^ vector (Fig. 1A). No significant alterations of the mTOR pathway effectors were observed in HEK293 cells transfected with the three GNAQ expressing vectors when compared to cells transfected with the empty vector (Fig. 1).

A significant increase in the level of pmTOR Ser2448 (*p* < 0.01 for BRAF^wt^ and BRAF^V 600E^) and pS6 Ser235/236 (*p* = 0.01 for BRAF^wt^ and *p* < 0.01 for BRAF^V 600E^) proteins was observed when comparing cells transfected with BRAF^wt^ and BRAF^V 600E^ vectors to cells transfected with the empty vector.

**Figure 2:**
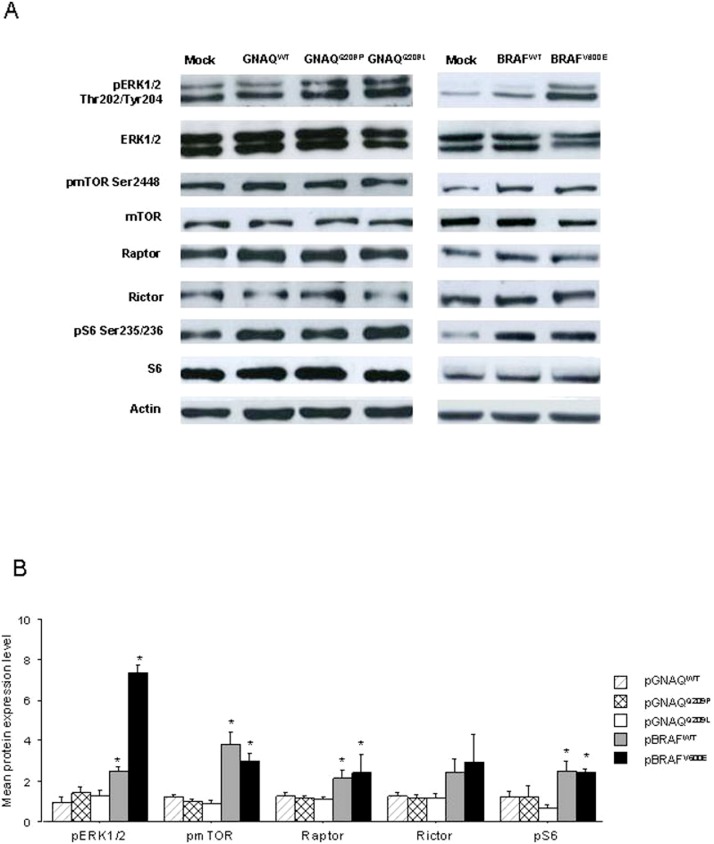
Graphic representation of the mean fold change of protein expression, observed in HEK293 cells, transfected with BRAF^wt^ and BRAF^V 600E^ expressing vectors compared to cells transfected with GNAQ^WT^, GNAQ^Q209P^ and GNAQ^Q209L^ expressing vectors, in at least three sets of experiments. Error bars are standard error. ∗ refers to a significant (*p* < 0.05) difference when comparing cells transfected with each BRAF vectors with all GNAQ vectors.

Comparing HEK293 cells transfected with GNAQ and BRAF vectors, a significantly higher expression of pERK1/2 (*p* = 0.02 for wt vectors and *p* < 0.01 for mutated vectors) and pmTOR (*p* = 0.01 for wt vectors and *p* < 0.01 for mutated vectors) was found in the cells transfected with BRAF vectors (Fig. 2). Cells transfected with BRAF^V 600E^ disclosed higher levels of raptor than cells transfected with GNAQ^Q209P^ and GNAQ^Q209L^ mutant vectors (*p* = 0.03). A higher pS6 expression was found in cells transfected with BRAF^V 600E^ than in cells transfected with GNAQ^Q209L^ vector (*p* = 0.05). Although not significant, we also found a tendency for higher pmTOR, raptor and rictor expression in cells transfected with BRAF^V 600E^ than in cells transfected with GNAQ^Q209L^ vector.

### Proliferation and apoptosis in cell lines transfected with BRAF and GNAQ vectors

No significant alterations were found either in proliferation (BrdU assay) or in apoptosis (TUNEL assay) when comparing HEK293 cells expressing BRAF and GNAQ vectors with cells transfected with the empty vectors (Fig. S1).

### Melanoma cell growth inhibition after treatment with RAD001

The genetic alterations in the mutational hot spot regions of BRAF, GNAQ and NRAS genes in the 8 cell lines were verified (Table 1).

**Table 1:** Summary of BRAF, NRAS, and GNAQ mutational status in melanoma cell lines.

Location	Cell line	Tissue	BRAF	NRAS	GNAQ
Skin melanoma cell lines	A375	Skin	V600E	wt	wt
	G361	Skin	V600E	wt	wt
	BLM	Lung metastasis	wt	wt	wt
	Mewo	Skin	wt	wt	wt
Uveal melanoma cell lines	92.1	Choroid	wt	wt	Q209L
	OMM2.3	Liver metastasis	wt	wt	Q209L
	Mel 285	Choroid	wt	wt	wt
	OMM1	Subcutaneous metastasis	wt	wt	wt

**Notes.**

wt = wild type.

**Figure 3:**
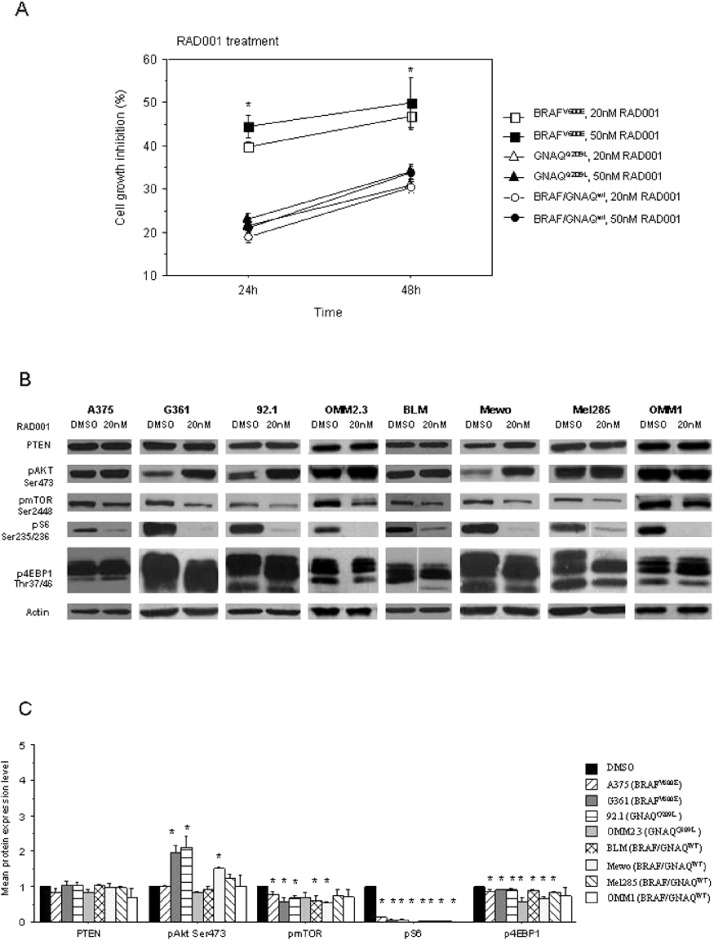
(A) Percentage of growth inhibition of melanoma cell lines treated with 20 nM and 50 nM of RAD001 for 24 and 48 h compared to non-treated cells in six sets of experiments. Error bars are standard error. ∗ = *p* < 0.05 (*BRAF* mutated cell lines *versus* all other cell lines); (B) representative Western blot analysis of PTEN, pAKT, pmTOR, pS6 and p4EBP1 expression observed in the melanoma cell lines after 20 nM RAD001 treatment for 24 h compared to non-treated cells; (C) graphic representation of the mean fold change of activated protein expression observed. Error bars are standard error; ∗ refers to a significant (*p* < 0.05) difference when comparing cells treated with 20 nM of RAD001 for 24 h compared to non-treated cells.

The effects of the mTOR pathway inhibitor RAD001 on melanoma cell growth in monolayer culture was determined by SRB assay (Skehan et al., 1990). Treatment of melanoma cells with 20 nM and 50 nM of RAD001 yielded variable growth inhibition in all the cell lines at 24 h and 48 h (Fig. 3A and Fig. S2). Cell lines harbouring BRAF mutation revealed to be more sensitive to RAD001 than the other cell lines tested, with growth inhibition rates of 40% and 44% at 24 h and 47% and 50% at 48 h, with 20 nM and 50 nM of RAD001, respectively, which are significantly higher than cell lines harbouring GNAQ mutations and cell lines wild type for both genes at 24 h (*p* < 0.01) and also at 48 h of treatment (*p* ≤ 0.01).

### Expression of mTOR pathway effectors in melanoma cell lines treated with RAD001

The efficacy of RAD001 in inhibiting the mTOR pathway was evaluated by Western blot analysis for PTEN, phosphorylated AKT at Ser473, mTOR at Ser2448, S6 at Ser235/236 and 4E-BP1 at Thr37/46AKTSer473. The results obtained at 24 h of treatment with 20 nM are presented in Fig. 3B. RAD001 effectively inhibits phosphorylation of S6 and partially inhibits phosphorylation of mTOR and 4EBP1 in all the evaluated cell lines. Phosphorylation of AKT was enhanced after treatment with RAD001 and no alteration was found in PTEN expression. Similar results were observed after 48 h of treatment with 20 nM and with 50 nM RAD001 in the two time points (data not shown).

### Expression of MAPK and mTOR pathways effectors in melanoma cell lines treated with U0126

**Figure 4:**
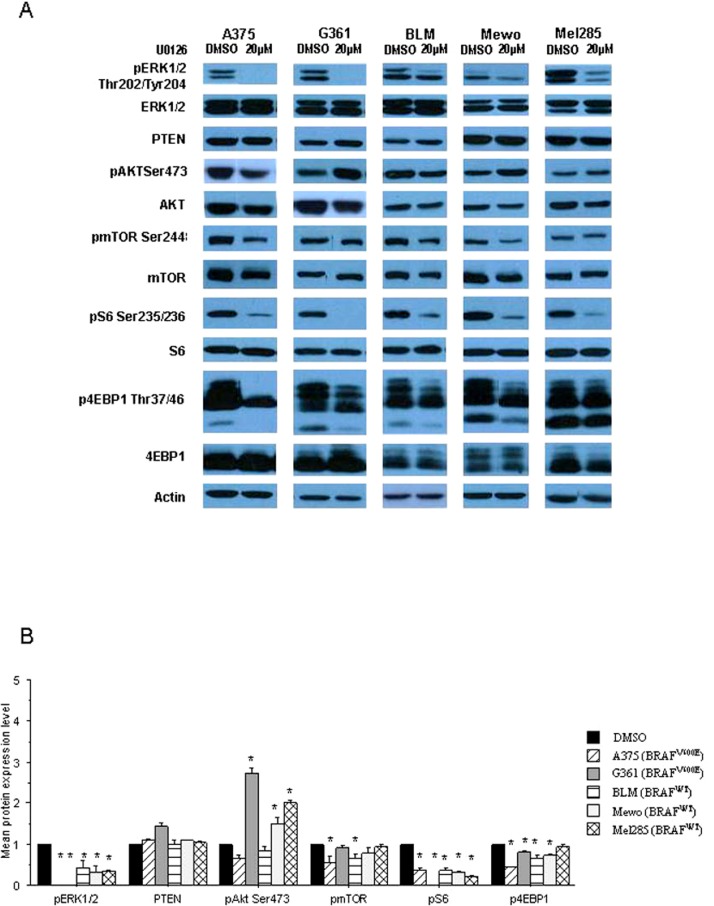
(A) Representative Western blot analysis of pERK1/2, PTEN, pAKT, pmTOR, pS6 and p4EBP1 expression observed in the melanoma cell lines after U0126 treatment compared to non-treated cells; (B) graphic representation of the mean fold change of activated protein expression observed. Error bars are standard error. ∗ refers to a significant (*p* < 0.05) difference when comparing cells treated with 20 µM of U0126 for 24 h compared to non-treated cells.

The efficacy of U0126 in inhibiting the MAPK pathway was evaluated by the levels of phosphorylated ERK1/2 at Thr202/Tyr204 and the efficacy in inhibiting the mTOR pathway was evaluated by analysis of PTEN, phosphorylated AKT at Ser473, mTOR at Ser2448, S6 at Ser235/236 and 4E-BP1 at Thr37/46. The results obtained at 24 h of treatment are presented in Fig. 4. U0126 effectively inhibits phosphorylation of ERK1/2 and also inhibits phosphorylation of mTOR, S6 and 4EBP1 in the evaluated cell lines. Phosphorylation of AKT was generally enhanced after treatment with U0126 and PTEN expression was not altered.

## Discussion

Constitutive MAPK activation seems to be a common event in both skin and uveal melanomas, although it occurs through different mechanisms: in skin melanoma, through BRAF and NRAS activating mutations (Reifenberger et al., 2004), and in uveal melanoma MAPK activation can occur through GNAQ activating mutations (Populo et al., 2011b; Van Raamsdonk et al., 2009).

In a previous report we described MAPK and AKT/mTOR pathway activations in a series of skin melanomas, where an association between BRAF mutation and high mTOR pathway activation was observed (Populo et al., 2011a). In thyroid carcinomas we also disclosed an association between BRAF mutation and mTOR pathway overactivation (Faustino et al., 2012). We have observed that BRAF over-expression lead to a significant increase in the expression level of pmTOR Ser2448 and pS6 Ser235/236 *in vitro* (Faustino et al., 2012).

Activation of MAPK and AKT/mTOR pathways was found in a series of uveal melanomas (Populo et al., 2010), but in those cancers this activation did not relate with the presence of GNAQ mutation (Populo et al., 2011b). Taken together, our previous reports suggest that BRAF activation, but not GNAQ activation, can enhance the activity of the mTOR pathway.

In the present work, GNAQ wild-type and mutant vectors lead to ERK1/2 activation, in accordance with the results reported by Raamsdonk et al., after transfection of hTERT/ CDK4^R24C^/ p53^DD^ melanocytes with GNAQ^Q209L^ vector (Van Raamsdonk et al., 2009). However, neither GNAQ wild-type nor mutated forms, lead to an increase in mTOR pathway activation, which is in line with the lack of association between GNAQ mutational status and mTOR pathway activation that we have reported in human uveal melanoma samples (Populo et al., 2011b) and also with the lack of alteration in AKT phosphorylation after loss of mutant GNAQ, already reported by others in uveal melanoma cell lines (Khalili et al., 2012). At variance, we observed that, besides ERK1/2 activation, wild-type and mutant BRAF lead to a significant increase in the expression level of pmTOR Ser2448 and pS6 Ser235/236 *in vitro*. The activation of mTOR pathway by BRAF is consistent with our previous studies on human tumours (Populo et al., 2011a).

We performed transient transfection with activated BRAF and GNAQ vectors and we did not find significant alterations in proliferation and apoptosis in the cell lines. Overexpression of BRAF^V 600E^ was previously reported to inhibit cell proliferation and induce senescence in long term experiments (Gray-Schopfer et al., 2006; Michaloglou et al., 2005), whereas GNAQ overexpression has no effect in cell growth (Van Raamsdonk et al., 2009). Further experiments (incorporating transformation of melanocytes and/or melanoma cell lines) will be necessary in order to verify if a sustained MAPK pathway activation, with or without concomitant mTOR pathway activation, may be sufficient to substantially modify proliferation and apoptosis in long term experiments. Alternatively, other pathways can also be involved in the proliferative burst in these tumours. Recently, Garcia-Marcos and co-authors reported that activating mutations in GNA01 gene, which also encodes a G-protein α*q*-subunit, enhances Stat3 activation (Garcia-Marcos, Ghosh & Farquhar, 2011). Concordantly, GNAQ wild-type and mutated forms also seem to drive higher expression of pStat3 Tyr705 (Fig. S3), which might mediate GNAQ oncogenic effects.

We found higher sensitivity to RAD001 treatment in the cutaneous melanoma cell lines harbouring a BRAF^V 600E^ mutation, in line with the higher expression of pmTOR and pS6 in cells transfected with BRAF^V 600E^. Although all the cell lines displayed a significant reduction of S6 phosphorylation, which is considered a marker of mTOR inhibition (O’Reilly & McSheehy, 2010), cell lines harbouring a BRAF^V 600E^ mutation (A375 and G361) displayed significant higher growth rate inhibition after RAD001 treatment than the other melanoma cell lines harbouring GNAQ^Q209L^ (92.1 and OMM2.3), or BRAF^wt^ and GNAQ^wt^ (BLM, Mewo, OMM1 and Mel285) cell lines. Similar results were reported by Ho et al. (2012) that observed higher sensitivity to mTOR and MEK inhibition in uveal melanoma cell lines harbouring BRAF mutations. In our work, we used cutaneous melanoma cell lines where BRAF mutations are the most common alteration and BRAF is considered an oncogene (Smalley & Herlyn, 2004). These data might support our previous suggestion that skin melanoma with BRAF mutation can be more sensitive to mTOR inhibition therapy (Populo et al., 2011a).

Not surprisingly, we verified that the inhibition of the mTOR pathway, by RAD001, and the MAPK pathway, by U0126, lead to AKT upregulation. This effect was previously reported and is supposed to occur through the abolishment of a S6K1/IRS-1 negative feedback and the induction of upstream receptor tyrosine kinase signalling (O’Reilly et al., 2006).

In the present study, uveal melanoma cell lines disclosed a significant reduction of S6 phosphorylation and growth inhibition after RAD001 treatment, although at a significantly lower level than the cell lines which harbour a BRAF^V 600E^ mutation. Therefore, we cannot definitively rule out the clinical potential of mTOR inhibition for the treatment of human uveal melanomas. Of note, Khalili et al., proposed that PI3K inhibition enhance the effects of MEK inhibition and the combination may be an effective therapy in uveal melanoma, particularly in a GNAQ mutant background (Khalili et al., 2012).

We observed that the abolishment of MAPK activity by U0126 treatment leads to mTOR pathway inhibition. A synergistic reduction of melanoma cell proliferation and induction of cell death with combined mTOR and MAPK pathway inhibition was already reported (Gopal et al., 2010; Lasithiotakis et al., 2008; Molhoek, Brautigan & Slingluff, 2005), suggesting that this combined inhibitory therapy may benefit patients with BRAF mutant melanomas.

It’s worth mentioning that no molecular markers are used in most of the clinical trials with mTOR inhibitors. Loss of PTEN and activation of AKT were suggested to be associated with increased tumour cell sensitivity to the mTOR inhibitors (Kurmasheva, Huang & Houghton, 2006), although only the loss of PTEN expression was used, in clinical trials, as a marker to evaluate glioblastoma sensitivity to rapamycin treatment (Cloughesy et al., 2008). Our results further support that BRAF, already used as a putative predictive marker of the effectiveness of MAPK pathway inhibition therapy (www.clinicaltrials.gov), might also be useful, in skin melanomas, as a predictive marker of mTOR pathway inhibition therapy alone or in combination with MAPK inhibitors.

## Conclusions

To the best of our knowledge, this is the first study comparing the cellular effects of two major oncogenic events, BRAF and GNAQ mutations, in melanomagenesis using both cutaneous and uveal models. Our results suggest that activated BRAF and GNAQ genes do not cause equivalent cellular effects and that only BRAF activation seems to lead to a higher activation of the mTOR pathway. Therefore, the activation of both genes seems to evolve through different pathways, reinforcing the concept that diverse pathogenic mechanisms drive the development of skin and uveal melanomas. Thus, strategies for melanoma therapy should consider the mutational status, and BRAF mutant melanomas may be more sensitive to mTOR inhibition therapy alone or in combination with MAPK inhibitors, such as vemurafenib, the BRAF^V 600E^ inhibitor already approved for the treatment of advanced melanoma (www.fda.gov).

## Supplemental Information

10.7717/peerj.104/supp-1 Figure S1 Representative images and graphic representation of the mean number of proliferative cells (A) and apoptotic cells (B) in HEK293 cells transfected with BRAF^wt^ and BRAF^V 600E^ expressing vectors, and with GNAQ^WT^, GNAQ^Q209P^ and GNAQ^Q209L^ expressing vectors, compared to cells transfected with an empty vector in at least three sets of experiments. Error bars are standard error.

10.7717/peerj.104/supp-2 Figure S2 Graphic representation of the percentage of growth inhibition of eight melanoma cell lines treated with 20 nM and 50 nM of RAD001 for 24 and 48 h compared to non-treated cells in six sets of experiments. Error bars are standard error.

10.7717/peerj.104/supp-3 Figure S3 Representative western blot analysis of pStat3 Tyr705 (Cell Signaling Technology) expression in HEK293 cells transfected with GNAQ^WT^, GNAQ^Q209P^ and GNAQ^Q209L^ expressing vectors compared to cells transfected with an empty vector. Error bars are standard error. ∗ refers to significant (*p* < 0.05) difference when comparing cells transfected with GNAQ vectors with those with the empty vector.
