# Supplementary figures and images for: GNAQ and BRAF mutations show differential activation of the mTOR pathway in human transformed cells
Source: PeerJ. 2013 Jul 23;1:e104. doi: 10.7717/peerj.104 (PMC3728761; doi:10.7717/peerj.104)

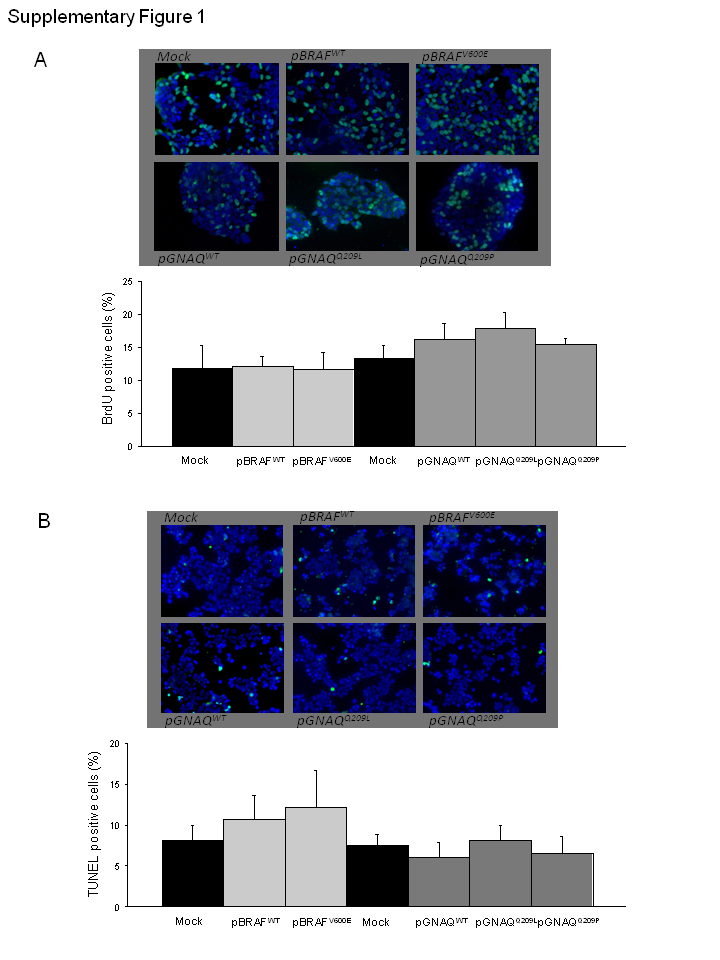

Supplement: Figure S1 — Representative images and graphic representation of the mean number of proliferative cells (A) and apoptotic cells (B) in HEK293 cells transfected with BRAFwt and BRAFV 600E expressing vectors, and with GNAQWT, GNAQQ209P and GNAQQ209L expressing vectors, compared to cells transfected with an empty vector in at least three sets of experiments. Error bars are standard error. [file peerj-01-104-s001.png]

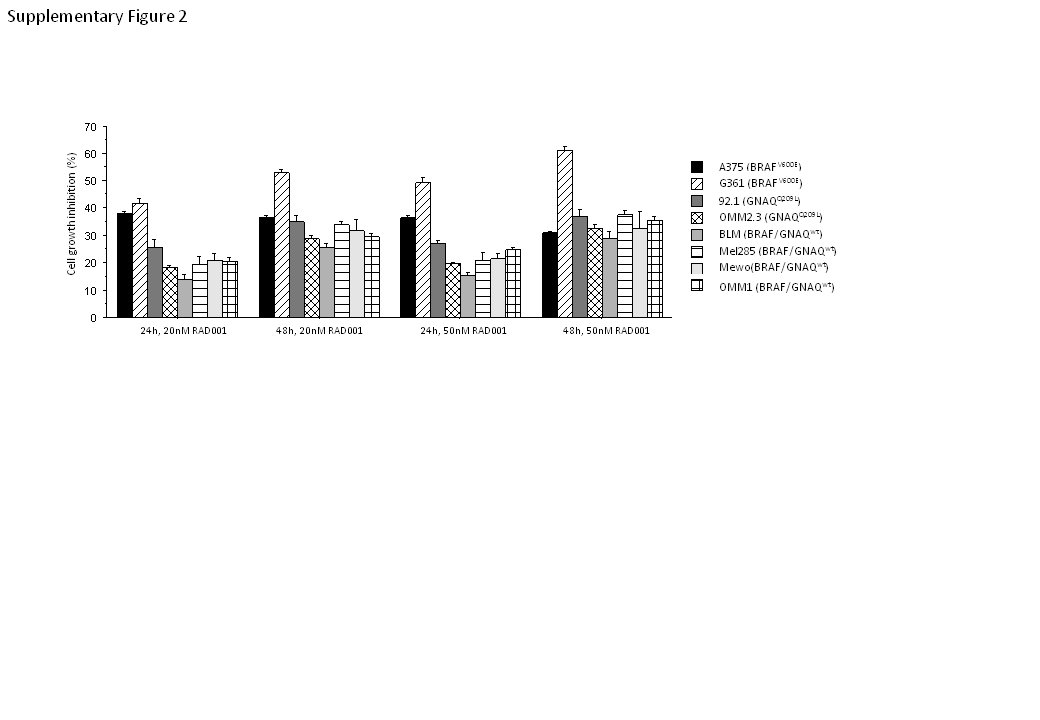

Supplement: Figure S2 — Graphic representation of the percentage of growth inhibition of eight melanoma cell lines treated with 20 nM and 50 nM of RAD001 for 24 and 48 h compared to non-treated cells in six sets of experiments. Error bars are standard error. [file peerj-01-104-s002.png]

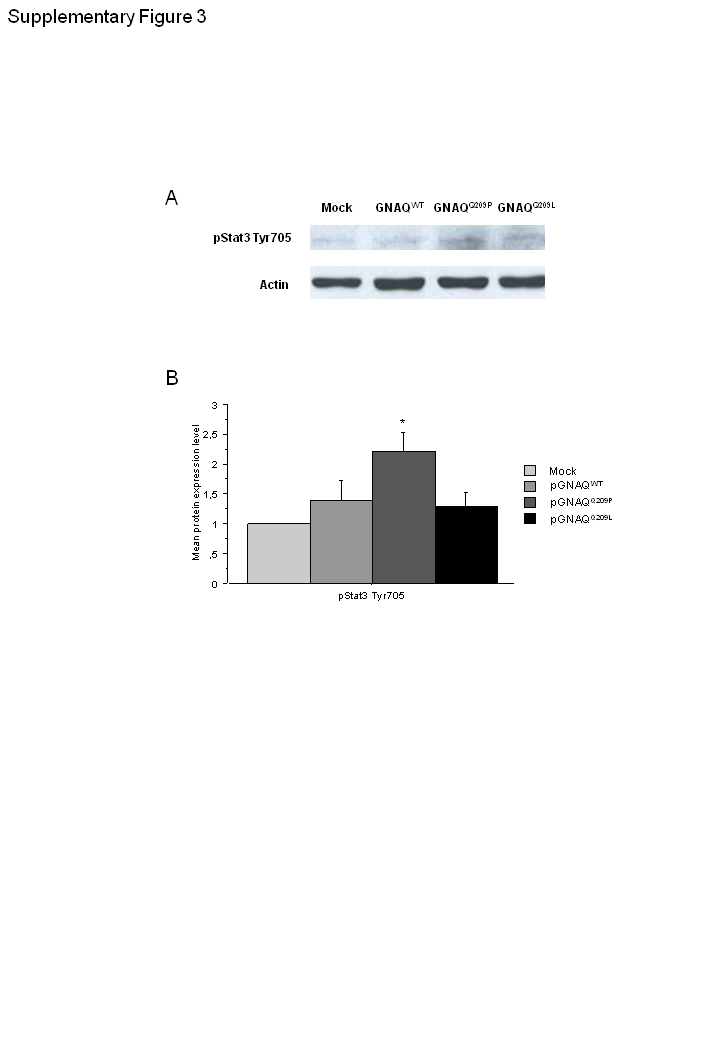

Supplement: Figure S3 — Representative western blot analysis of pStat3 Tyr705 (Cell Signaling Technology) expression in HEK293 cells transfected with GNAQWT, GNAQQ209P and GNAQQ209L expressing vectors compared to cells transfected with an empty vector. Error bars are standard error. ∗ refers to significant (p < 0.05) difference when comparing cells transfected with GNAQ vectors with those with the empty vector. [file peerj-01-104-s003.png]
